# Snow Microorganisms Colonise Arctic Soils Following Snow Melt

**DOI:** 10.1007/s00248-023-02204-y

**Published:** 2023-03-20

**Authors:** Lucie A. Malard, Benoit Bergk-Pinto, Rose Layton, Timothy M. Vogel, Catherine Larose, David A. Pearce

**Affiliations:** 1 Faculty of Health and Life Sciences, Northumbria University, Newcastle-Upon-Tyne, NE1 8ST UK; 2 Department of Ecology and Evolution, University of Lausanne, 1015 Lausanne, Switzerland; 3 Environmental Microbial Genomics, Laboratoire Ampère, École Centrale de Lyon, CNRS, University of Lyon, Lyon, France; 4 BioIT, TAG (Transversal Activities in Applied Genomics) Sciensano, 1050 Brussels, Belgium

**Keywords:** Microbial colonisation, Airborne dispersal, Coalescence, Snow, Soils, Arctic ecosystems, Bacterial diversity

## Abstract

Arctic soils are constantly subjected to microbial invasion from either airborne, marine, or animal sources, which may impact local microbial communities and ecosystem functioning. However, in winter, Arctic soils are isolated from outside sources other than snow, which is the sole source of microorganisms. Successful colonisation of soil by snow microorganisms depends on the ability to survive and compete of both, the invading and resident community. Using shallow shotgun metagenome sequencing and amplicon sequencing, this study monitored snow and soil microbial communities throughout snow melt to investigate the colonisation process of Arctic soils. Microbial colonisation likely occurred as all the characteristics of successful colonisation were observed. The colonising microorganisms originating from the snow were already adapted to the local environmental conditions and were subsequently subjected to many similar conditions in the Arctic soil. Furthermore, competition-related genes (e.g. motility and virulence) increased in snow samples as the snow melted. Overall, one hundred potentially successful colonisers were identified in the soil and, thus, demonstrated the deposition and growth of snow microorganisms in soils during melt.

**Supplementary Information:**

The online version contains supplementary material available at 10.1007/s00248-023-02204-y.

## Introduction

The global invasion of organisms is a major threat to ecosystems and the associated biota [1]. For decades, research has focused on macroorganisms’ invasions [2, 3] with only recent interest on microbial invasions [4–6]. Recent investigations on soil microbial communities have shown clear biogeographic patterns [7–9] with some taxa presenting restricted distributions, highlighting the potential for endemicity and, as a result, the susceptibility of ecosystems to invasions [6, 10–13]. In the Arctic, aerial transport is the primary source of new biological inputs. Microbiological invasions have been shown to occur in laboratory microcosms mimicking Arctic conditions [14], but the impacts of such invasions on natural resident communities are unclear.

Microbial colonisation consists of four sequential processes: the introduction of the invader, establishment, growth and spread, and the impact of the coloniser on the resident microbial community [4, 5]. A successful colonisation depends on the adaptability of the incoming taxon to the new habitat and the resistance to invasion from the colonised habitat [4, 5, 15]. Successful colonisers first have to adapt and survive local environmental conditions, which may not be favourable compared to their ecosystem of origin; then, they have to compete with the resident community for resources in the new ecosystem [4, 16]. Therefore, their invasive potential depends on effective adaptations increasing the likelihood of survival and competitive advantage. Generally, high growth rates, dispersal ability, phenotypic plasticity, and genetic diversity increase the chance of success of the invader [17]. The ability to exploit empty niches would also increase the chance of perennial colonisation. Key genes involved in the adaptation to local environmental conditions include genes associated with the stress response such as oxidative stress or heat shock, but also the cell wall structure or capsule synthesis [18, 19]. Genes increasing competition potential include antibiotic production or resistance, increased motility, or better resource utilisation [19, 20]. Being an r-strategist with high growth rates and short generation time is also hypothesised to increase the chances of successful colonisation [17, 21–23], although, in extreme environments such as cold or nutrient limited systems, K-strategists may be better suited to tolerate the biotic/abiotic stressors and thus, more likely to successfully colonise. The chances of successful colonisation also depend on the resident microbial community and resource availability. For instance, high richness, diversity, and evenness decrease the chances of colonisation in temperate soils [15, 24–28]. On the other hand, resource pulses, where a peak in nutrient availability occurs over a short period of time, increase the chances of successful colonisation by increasing resource availability and decreasing competition [16, 26].

Natural communities are constantly subjected to microbial invasions from a wide variety of sources. In the Arctic, these are generally limited to either airborne, marine, or animal sources. Aeolian transport of microorganisms, where organisms are aerosolised, transported in the air, and deposited in new environments, is the most universal dispersal pathway [29, 30]. By characterising and comparing microbial communities living in the air and in the ecosystem of interest, the potential for successful dispersal and colonisation can be inferred by assessing similarities in diversity [31–34]. However, in the case where similarities are identified, whether the airborne microorganisms common to the ecosystem of interest might have travelled from distant habitats or have been aerosolised from the ecosystem of interest in the first place is difficult to determine.

Snow melt is a model that can be used to understand microbial colonisation in general and in the context of airborne dispersal [14]. Snow covers a minimum of 2 × 10^6^ km^2^ and a maximum of 45 × 10^6^ km^2^ of the Northern Hemisphere every year [35]. Snow influences the global climate through the albedo [36], but also locally through the insulation of soils to ambient environmental and climatic conditions [37]. In addition, the snow cover isolates the underlying soil from contact with microbes from outside sources for up to 10 months a year, as is the case in Svalbard [38]. Pristine snow is primarily seeded from the atmosphere and unique microbial communities develop within the snow over time [39–41] and actively participate in nutrient cycling [42]. Once the snow starts melting, the run-off travels vertically on flat terrain to reach the frozen soil layer and infiltrate the soil when no ice layer exists [40, 43, 44]. Although the percolation rate will vary depending on local conditions, snow microorganisms inevitably encounter soil microbial communities at the end of the snow melt. The snowpack is a source of potential colonisers as it contains between 10^2^ and 10^4^ microbial cells per mL of melted snow [45–47]. Snow melt also creates a peak in nutrient and solute availability in soils [40, 48–50]. This resource pulse may facilitate the colonisation of soils by snow microorganisms. In the case of soils where the community is rich and diverse and colonisation may generally be difficult [15], the snow melt may be an opportunity for snow microorganisms to establish in a new habitat.

Using snow melt as a model for dispersal and deposition of microorganisms in soil, the aim of this study was to determine the colonisation potential of snow microorganisms by monitoring microbial communities of the melting snow and underlying soil during melt. We used 16S rRNA gene amplicon sequencing to detect potential colonisers and observe shifts in community structure and shallow shotgun metagenome sequencing to assess functional potential and identify genes that might favour colonization.

## Material and Methods

### Sample Collection and Processing

**Fig. 1:**
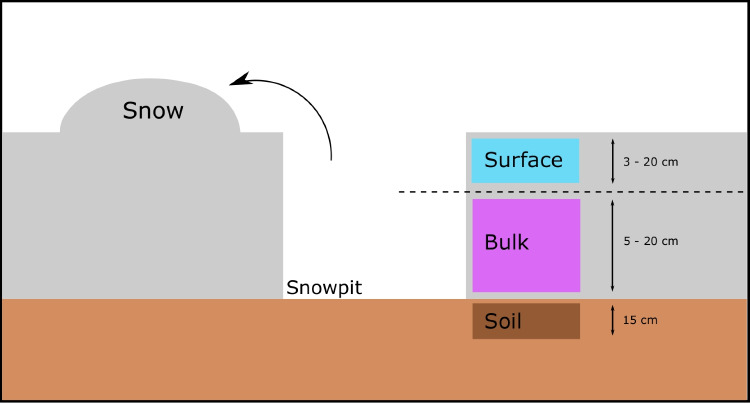
Diagram of the sampling design. A snowpit was dug, and surface, bulk, and soil samples were collected. At each sampling event, a new pit was dug in a previously undisturbed area. The thickness of the surface and bulk snow layer fluctuated with snow melt and precipitation events

Samples were collected during the snow melt in spring 2018 in Ny-Ålesund (78°56′N, 11°52′E), Svalbard (Fig. S1). The field site consisted of a 100 m^2^ perimeter, close to the laboratory of the AWIPEV research station, situated on the far south-end of the Ny-Ålesund village and bordered by Kongsfjorden, a 26-km-long and 3- to 8-km-wide fjord on the westcoast of Spitsbergen. Snow and soil sampling was conducted twice a week during the melt season, between May 1st and May 19th. Wearing Tyvek full body suits and gloves to avoid snow contamination, we dug snow pits using a sterilised Teflon shovel (Fig. 1) and collected snow and soil samples in 3-L sterile Whirl–Pak bags (Nasco, Fort Atkinson, WI, USA). Given that the snowpack underwent physical metamorphosis during the sampling period, the different snow layers changed over time. Snowpack stratigraphy could be assessed visually and by conducting hardness profiles. The surface layer was defined as the top layer of the snowpack (layer in contact with the atmosphere with the same hardness). The depth of the surface layer varied from 20 to 3 cm depending on the sampling date was generally composed of loosely packed snow and was exposed to the constant deposition of airborne microorganisms. The bulk snow samples constituted the underlying layers with a different hardness but were considered here as a single layer. Once at the bottom of the snow pit, 50 g of the underlying soil was collected within the first 15 cm using a sterilised trowel and 750 mL Whirl–Pak bags (Nasco) (Fig. 1). Overall, 6 sampling days with three sample types (surface snow, bulk snow, soil) were collected for a total of 18 samples (Table S1). Snow samples were processed immediately after collection. Samples were left to melt at room temperature and filtered onto nitrocellulose 0.22 µm filters (Merck Millipore, Germany) using a sterile filtration unit (Nalge Nunc International Corporation). The resulting filters were placed in sterile falcon tubes and stored at − 20 °C. Soil samples were stored at − 20 °C after collection.

### Weather Conditions During Snow Melt

**Fig. 2:**
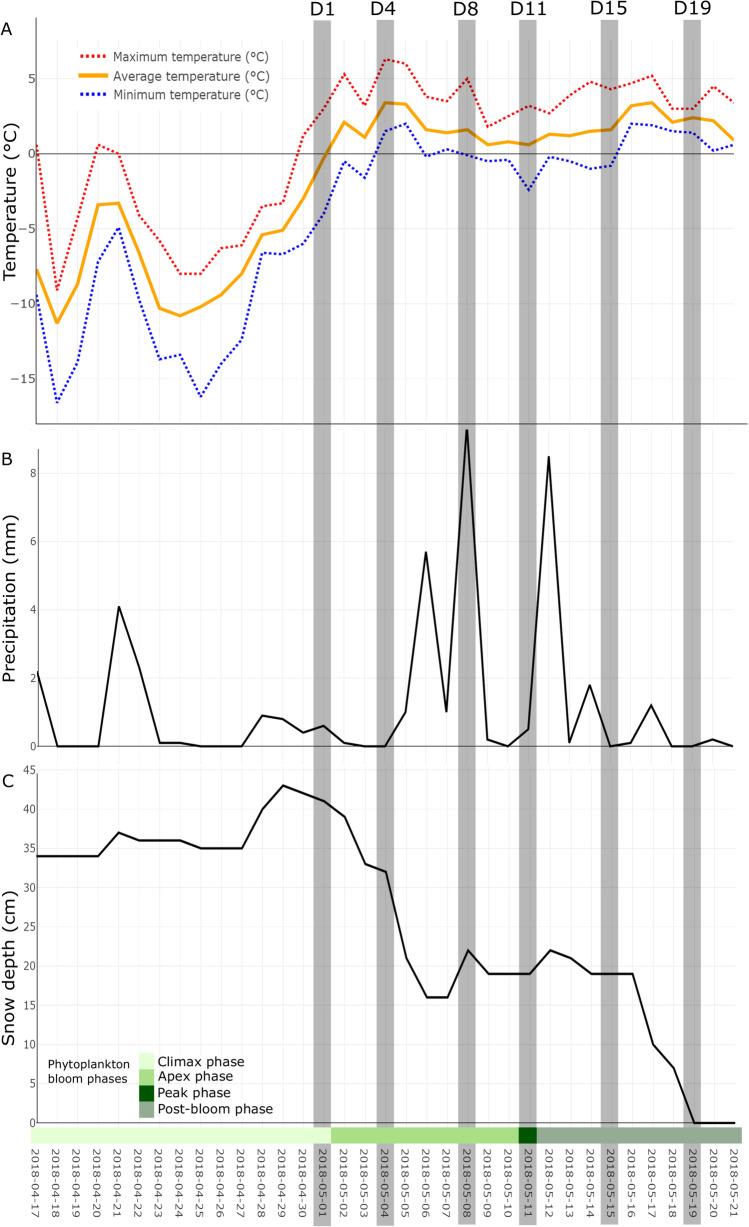
Ny-Alesund weather information before and during the sampling period for (**A**) air temperature (°C), (**B**) precipitation (mm), and (**C**) snow depth (cm). Grey areas indicate sampling days. The different phases of the phytoplankton bloom in Kongsfjorden are based on observations from Feltracco et al. [52]

The temperature (Fig. 2A), precipitation (Fig. 2B), and snow depth (Fig. 2C) variation were extracted from eKlima for the sampling period and 14 days prior. We sampled at six different times during the snowmelt: May 1st also known as day 1 (D1) was the baseline community where no major changes in snow conditions had occurred yet. Temperatures were below 0 °C, precipitation was low, and the snowpack was stable at 34 cm depth and up to 43 cm on April 29th. April 30th was the first day with temperatures above 0 °C, but the snow melt began on May 2nd when average temperatures reached 2.1 °C. Thus, at day 1 (D1), we sampled a stable snowpack. We also sampled on May 4th (D4), May 8th (D8), May 11th (D11), May 15th (D15), and May 19th (D19), which was the last day of the snow melt. We observed a peak of snow melt between day 4 and day 6, where 50% of the snowpack melted in 48 h and the snow depth went from 32 to 16 cm. This coincided with a peak in temperatures to over 6 °C. The snowpack stabilised between day 8 and day 16, with an increase in precipitation and average temperatures around 1 °C. The second peak in snow melt occurred between day 16 and day 19, with maximum temperatures reaching over 5 °C. This led to the complete melt of the remaining snowpack. May 19th was the last sampling day (D19) when the remaining snowpack measured 7 cm deep and melted by the end of the day. As the snowpack melted, the sampled layer thickness decreased over time. However, because of the changing density, the volume of melted snow (melt water) filtered remained relatively stable, around 1500 mL per sample, and, therefore, should have a minimal impact on the presented results. We should note that a study monitoring airborne microbial communities during the same time period recorded an algal bloom in Kongsfjorden [51], with the peak of the bloom on May 11th (Fig. 2).

### DNA Extraction, Shallow Shotgun Metagenomic Sequencing, 16S rRNA Amplicon Sequencing, and Bioinformatic Processing

Soil DNA was extracted from 0.25 g of each sample using the PowerSoil kit (Qiagen, Carlsbad, CA, USA), while snow DNA was extracted from the filters using the PowerWater kit (Qiagen) and following the manufacturers’ protocol. The metagenomic library was prepared using the Nextera XT DNA library preparation kit (Illumina, CA, USA) and following the manufacturers’ protocol. Libraries were cleaned using the AMPure XP beads (NEB) and were pooled and loaded on the MiSeq for sequencing. The adaptors were removed from the fastq files using cutadapt [53] and merged in QIIME [54] using FLASH [55]. Merged fastq files were uploaded on MG-RAST [56] and subjected to quality control, which included dereplication, removal of human related sequences, removal of ambiguous bases (> 5), and a quality phred score > 15. Once all metagenomes were processed on the MG-RAST server, reads were taxonomically annotated based on the NCBI RefSeq database (Pruitt et al., 2006) with a maximum e-value of 10^−5^, 80% identity, 30 bp overlapping length, and a minimum abundance of 5 reads. Reads were also functionally annotated by similarity searching against the Rapid Annotation using Subsystem Technology (RAST) database [57], with a maximum e-value of 10^−5^, 60% identity, 15 bp overlapping length, and a minimum abundance of 5 reads. The RAST subsystem classification assigns genes to functional roles, themselves grouped into subsystems. The taxonomic and gene abundance tables were analysed in R.

16S rRNA gene libraries were constructed using the universal primers 515F and 806R [52] to amplify the V4 region. Amplicons were generated using a high-fidelity Accuprime DNA polymerase (Invitrogen, Carlsbad, CA, USA), purified using AMPure magnetic bead capture kit (Agencourt, Beckman Coulter, MA, USA), and quantified using a QuantIT PicoGreen fluorometric kit (Invitrogen). The purified amplicons were pooled in equimolar concentrations using a SequalPrep plate normalization kit (Invitrogen), and the final concentration of the library was determined using a SYBR green quantitative PCR (qPCR) assay. Libraries were mixed with Illumina-generated PhiX control libraries and our own genomic libraries and denatured using fresh NaOH and sequenced on the Illumina MiSeq V2 (500 cycles). The resulting amplicons were processed using the DADA2 pipeline [58]. Forward and reverse read pairs were trimmed and filtered, with forward reads truncated at 230 base pairs (bp) and reverse reads at 200 bp, no ambiguous bases allowed, and each read required to have < 2 expected errors based on their quality scores. Amplicon sequence variants (ASVs) were independently inferred from the forward and reverse reads of each sample using the run-specific error rates. Reads were dereplicated, pairs were merged, and chimeras were removed. Taxonomic assignment was performed against the SILVA v128 database [59, 60] using the implementation of the RDP (ribosomal database project) naive Bayesian classifier [61]. It resulted in a total of 490,185 reads (27,235 reads/sample on average), assigned against 6934 ASVs.

### Statistical Analyses

All statistical analyses were performed in the R environment using primarily a combination of the vegan [62] and phyloseq [63] packages, and figures were made with ggplot2 [64]. Weather information was extracted from the Norwegian Meteorological Institute eKlima database [65], which records and makes available daily weather information, including temperature, precipitation, and snow depth. The taxonomic and functional tables were extracted from MG-RAST and normalised by the number of reads of each sample to account for variation in read number (total sum scaling) and used to assess differences in composition and function using principal coordinate analysis (PCoA). Linear models were built to investigate changes in community and functional composition with time. We used linear models to investigate changes in relative abundance of functions of interests (related to colonisation potential) with snow melt; only genes categories presenting some variations were retained.

Amplicon sequencing of bacterial communities was conducted to identify potentially colonising taxa. As it focuses on one group of organisms, the depth provides a more detailed insight into the community. However, due to the low biomass of the snow samples and higher potential for contamination during amplicon sequencing, the prevalence function from the decontam package [66] was used to remove contaminants. We had three extraction kit controls and three negative MiSeq controls to use for decontam. We ran the prevalence function with a threshold of 0.5 to identify as contaminants all sequences that were more prevalent in negative controls than in positive samples and identified 54 potential contaminants that were removed from further analyses.

Bacterial diversity was calculated using phyloseq, and, as for metagenomics, the table was normalised by total sum scaling to investigate community composition. To identify potential colonisers, we removed all ASVs uniquely present in the snow (surface and bulk) and those uniquely present in the soil. We also removed any ASV identified in the soil on the first day of sampling or first identified in the soil. While the absence of taxa may reflect the biases associated with molecular techniques or detection limit, the sequencing depth of each sample should have been sufficient, especially as the first and last soil samples had the deepest sequencing depth (Fig. S2). The remaining 596 ASVs were considered potential colonisers: absent from the soil on the first day of sampling but identified at least once at later stages and always identified in the snow. From these, colonisers were considered potentially successful colonisers if they were identified in at least two soil samples and were still present on the last day of sampling. Finally, based on the existing literature [22, 67, 68], potentially successful colonisers were classified by their most likely life strategy, either copiotrophs (r-strategists), oligotrophs (K-strategists), or unclear when no information was found in the literature (Table S2). We should note that some microbial phyla, such as Bacteroidetes or Actinobacteria, have been classified as both copiotrophs and oligotrophs in the literature. As a result, we selected the most common/recent classification found in the literature with this caveat in mind.

### Data Availability

The metagenomes used for this manuscript are deposited on MG-RAST under the accession project number MPG89221 (Table S1). The amplicon sequences used in this manuscript are deposited at the European Nucleotide Archive under the BioProject accession number PRJNA564220.

## Results

### Microbial Communities Change with Snow Melt (Shotgun Metagenomics)

**Fig. 3:**
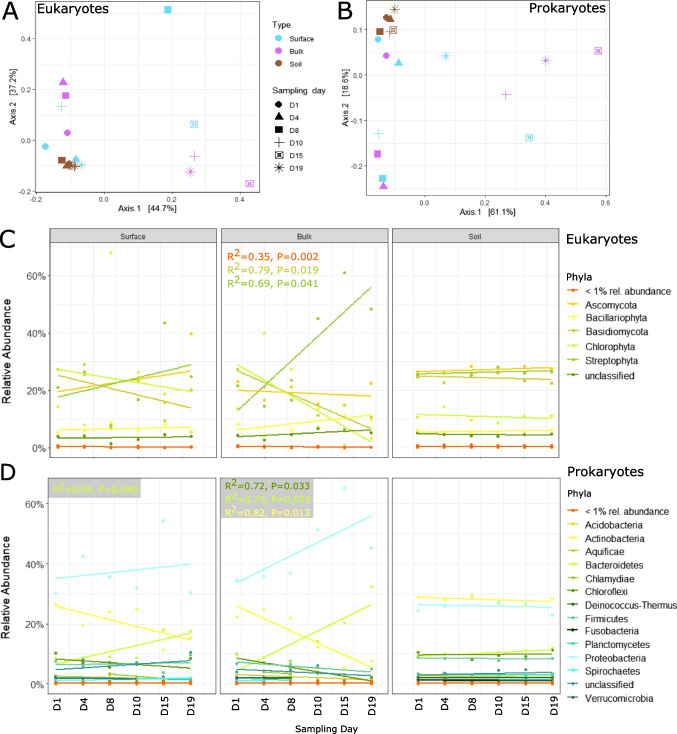
Eukaryotic and prokaryotic communities assessed via shotgun metagenomic sequencing. Significant (*p* < 0.05) linear regressions are displayed. **A** PCoA of the eukaryotic communities (Bray–Curtis dissimilarity) showing the stability of soil communities, the two clusters of bulk snow, and the variability of surface snow communities. **B** PCoA of the bacterial communities (Bray–Curtis dissimilarity) showing the stability of soil communities, the two clusters of bulk snow, and the variability of surface snow communities. **C** Eukaryotic community composition at the phylum level with phyla representing less than 1% of the community clustered together. **D** Bacterial community composition at the phylum level with phyla representing less than 1% of the community clustered together

Overall, bacteria and eukarya dominated all the communities examined while archaea, viruses, and other sequences were virtually absent from the metagenomic dataset (Fig. S3). Putative eukaryotic sequences represented approximately 30% of the sequences across sampling days (Fig. S3). Surface snow eukaryotic community composition showed high variability, with communities on day 8 and day 15 clustering away from other days (Fig. 3A). Sequences annotated as Ascomycota (fungi), Basidiomycota (fungi), Streptophyta (green algae and plants), and Chlorophyta (green algae) largely dominated the dataset. In surface snow, the overall increase in Streptophyta and Ascomycota was associated to a decrease in Chlorophyta and Basidiomycota (Fig. 3C). Bulk snow communities formed two distinct clusters: the first three and the last three sampling days (Fig. 3A). These were characterised by the significant increase in Streptophyta (Charophyceae) from day 11 (Fig. 3C), the day of the phytoplankton bloom peak in Kongsfjorden. The relative abundance of Ascomycota remained relatively stable over time, while Basidiomycota and Chlorophyta relative abundances decreased over time (Fig. 3C). In comparison, the soil eukaryotic community was more stable, with Ascomycota, Basidiomycota, Chlorophyta, and Streptophyta all well represented (Fig. 3A, C).

Bacterial sequences represented approximately 70% of the sequences (Fig. S3) of the metagenomic dataset. As for the eukaryotes, the bacterial communities displayed high variability (Fig. 3B). Actinobacteria decreased over time, while Bacteroidetes significantly increased over time, driven by the increase in Flavobacteriia (Fig. 3D). In bulk snow, the community composition formed the same two clusters as the eukaryotic community (Fig. 3B). A shift in the community composition was observed from day 11 onward, driven by the sudden increase in the relative abundance of Betaproteobacteria and Bacteroidetes (Flavobacteria) (Fig. 3D). All other taxa decreased after this shift (such as Actinobacteria, Chloroflexi, and Firmicutes), and some almost disappeared from the taxonomic profile (such as Planctomycetes and Verrucomicrobia). In the soil, no major shifts in diversity nor in community composition were observed (Fig. 3B, D).

### Changes in the Functional Profile of Microbial Communities with Snow Melt (Shotgun Metagenomics)

**Fig. 4:**
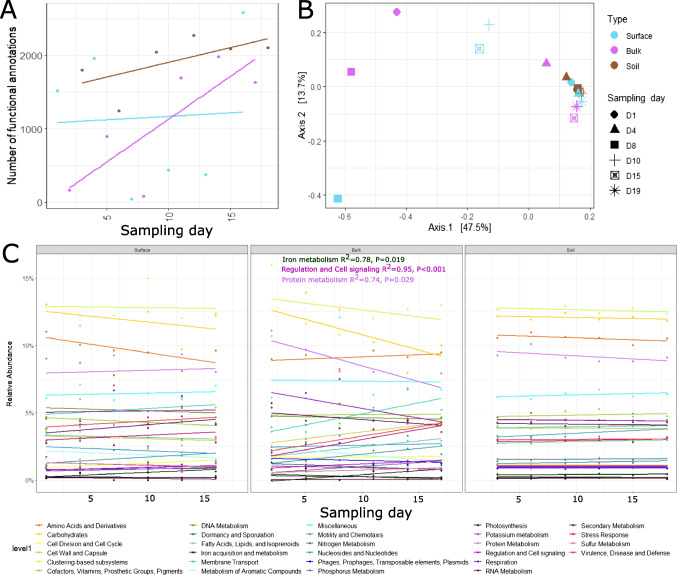
Functional profile of communities assessed via shotgun metagenomic sequencing. Significant (*p* < 0.05) linear regressions are displayed. **A** The number of functional annotations at each sampling day. **B** PCoA of the functional profile (Bray–Curtis dissimilarity) showing the stability of soil communities, and the variation of the snow profiles. **C** Functional profile of each sample at level 1 of the RAST subsystems

The number of functional annotations increased in the snow over time but was stable in soil samples (Fig. 4A). The PCoA of the functional profile using the RAST subsystems highlighted some variations from one sample to the next without any clear clustering of the snow samples. On the other hand, soil samples and some of the snow samples clustered closely together, illustrating a similar functional profile over time (Fig. 4B). Overall, the most abundant subsystems identified across all sample types and sampling days were related to cell function and metabolism, such as amino acid, carbohydrate, fatty acid, or protein metabolism, and to proteosomes and ribosome function within the ‘clustering-based subsystem’ (Fig. 4C). The relative stability of housekeeping genes over time was a good indicator that any functional changes observed were likely the result of ecological changes, such as variations in environmental conditions, rather than the influence of the number of sequencing reads. In both, surface and bulk snow, we observed changes in genes (although not always significant) associated with iron acquisition, protein metabolism, membrane transport, motility, virulence, and stress response associated genes. Some of these genes are likely to influence the colonisation potential by playing a role in competition, adaptation, and survival.

**Fig. 5:**
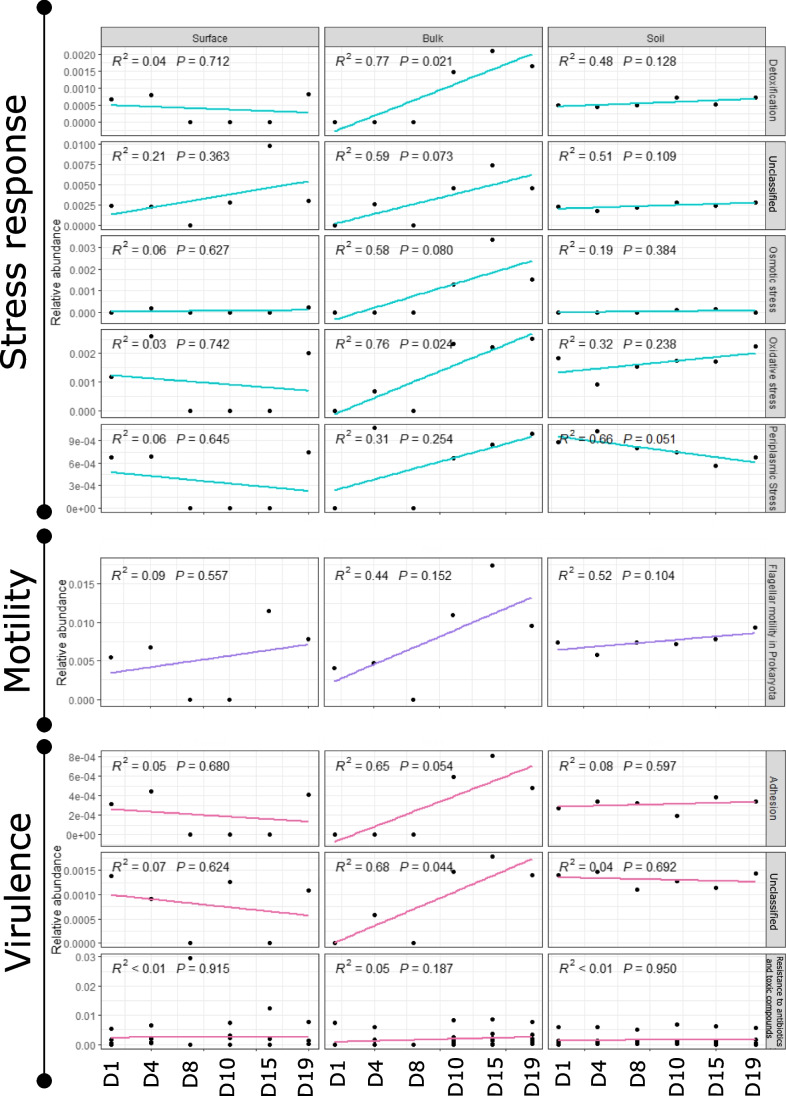
Linear models based on the relative abundance of selected functional genes likely playing a role in colonisation and illustrating changes with sample type and sampling day

For example, the relative abundance of genes associated with the stress response represented almost 5% of the functional profile and increased over time in bulk snow samples (Fig. 4C). Most changes in stress response genes were observed in bulk snow samples, with the increase of each category with time (Fig. 5). We identified an increase in genes protecting against oxidative stress, especially bacterial haemoglobins, and glutathione-related genes. We also identified ectoine and betaine biosynthesis genes, both compounds are used to protect against drought, salinity, temperature, or osmotic stress [69]. The relative abundance of these genes increased in bulk snow microorganisms at the end of snow melt. Genes associated with motility and chemotaxis represented approximately 2% of the functional profile and showed an increase in flagellar motility associated genes over time in bulk snow microorganisms (Fig. 5). At the level 1 of subsystem classification, genes associated with virulence, disease and defence accounted for approximately 5% of the profile and were primarily classified as ‘resistance to antibiotics and toxic compounds’ (Fig. 4C). Genes associated with cobalt-zinc-cadmium resistance were the most abundant and were identified in all samples. Besides environmental resistance genes, antibiotic resistance genes were also common, and the most abundant was the multidrug resistance efflux pump, which was present in all samples. Genes associated with the resistance to fluoroquinolones and beta-lactamase were primarily observed in soils and were stable over time while, in snow samples, their relative abundance fluctuated over time (Fig. 5). We also observed an increase in adhesion genes in bulk snow samples.

Other genes relevant to colonisation included genes associated with dormancy and sporulation although they represented < 1% of the functional profile and changes over time were not observed. Similarly, genes associated with the regulation and cell signalling represented approximately 1% of the functional profile while quorum sensing, and biofilm formation related genes were virtually absent from the profile (Fig. 4C).

### Bacterial Colonisation of Soils During Snow Melt (Amplicon Sequencing)

**Fig. 6:**
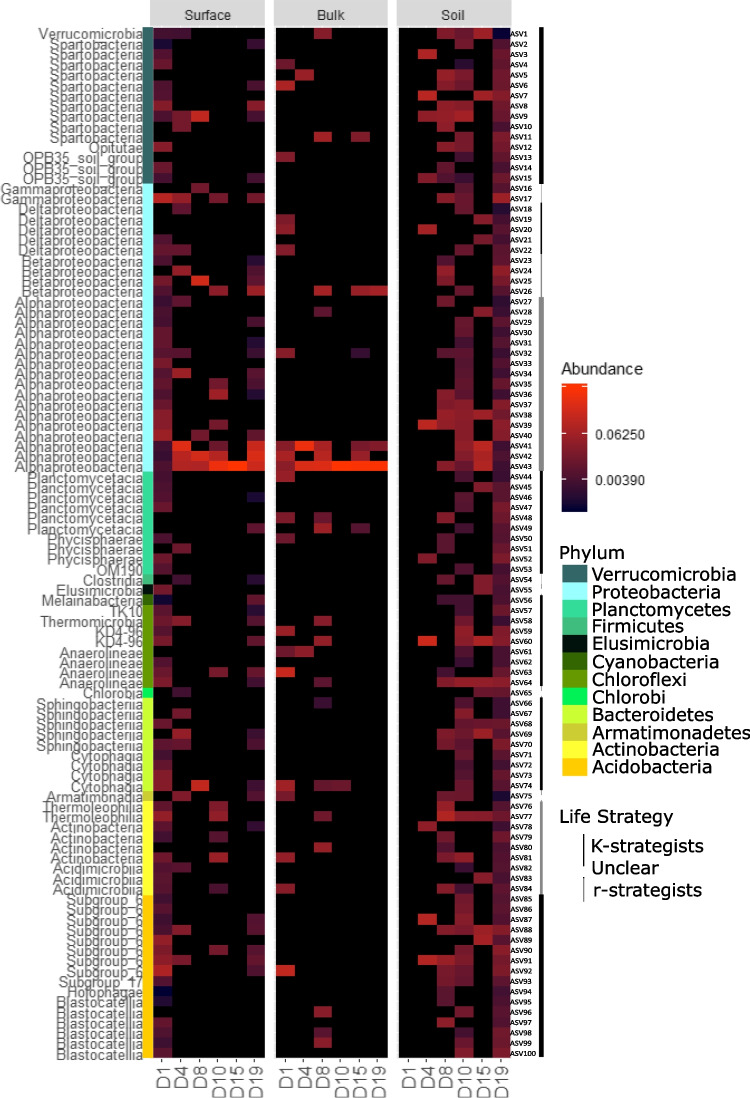
Heatmap of the relative abundance of potentially successful colonisers where each row corresponds to an ASV ordered by class. The coloured bar indicates the taxonomy at the phylum level

In addition to shotgun metagenomics, we assessed the bacterial communities using 16S rRNA gene amplicon sequencing to identify potential bacterial colonisers. Overall bacterial diversity decreased over time in snow samples (linear model: *R*^2^ = 0.79, *p* = 0.018) (Fig. S4). The patterns of bacterial diversity and community composition using 16S were similar to the metagenomics results, with the same clusters identified in the PCoA (Fig. S4B). The changes in snow community composition were more striking using the 16S, especially in the bulk snow samples. Overall, there was a significant increase in Proteobacteria and decrease in all other phyla (Fig. S4C). The soil community remained stable overall. Using the amplicon data, we identified 596 ASVs considered as potential colonisers. They were first identified in the snow and subsequently observed in the soil at least once. Of these, 100 ASVs were considered potentially successful colonisers as they were identified in at least two soil samples and were still present on the last day of sampling (Fig. 6). We classified these ASV by their most likely life strategy [67, 68] and found that 65 of the 100 potentially successful colonisers were considered oligotrophs (K-strategists) (Fig. 6, Table S2).

## Discussion

In this study, microbial communities in the snowpack and the soil were monitored to evaluate the colonisation potential. This was the first study to characterise microbial communities during snow melt and to monitor changes in the underlying soil community during snow melt in the field.

Based on the weather data, the snow melt lasted 19 days and represented a period of intense change for the microbial communities living in those ecosystems and was likely reflected in the taxonomic and functional profiles. Disturbances to the ecosystem have been shown to facilitate microbial colonisation [70], and therefore, the snow melt may be an opportunity for invading microorganisms to colonise the soil system. Furthermore, the snow melt has been previously characterised as a pulse in nutrient availability [40, 48–50], and although nutrient fluxes were not measured, resource pulses have been shown to facilitate colonisation in temperate soils [26, 71, 72].

### Invading Community

Due to the important role of snow cover on the climate by reflecting solar radiation (albedo) and the negative role of snow algae darkening the snow and decreasing the albedo [73, 74], a number studies have investigated and described eukaryotic communities in Arctic snow with a focus on algal communities [75–78]. In this study, Chlorophyta and Streptophyta dominated the algal community with variable abundances over time. These taxa are commonly identified in Arctic snow [76, 77], and the abundance peaks after the onset of snow melt are characteristic of algal blooms [74]. Snow algae are essential to the ecosystem as they actively fix carbon and become sources of organic carbon available for heterotrophic organisms [74, 79]. This increase in available organic carbon may be reflected by the increase of bacterial r-strategists (which grow faster in presence of available carbon) in the communities following the putative algal bloom [21, 67]. Many Betaproteobacteria are often considered r-strategists with fast growth rates [67] and have been associated with algal blooms [77, 80]. The genus *Massilia*, from the family Oxalobacteraceae, was responsible for the sudden increase in Betaproteobacteria. Members of the *Massilia* genus are heterotrophic, aerobic, motile, mesophile with a lower growth limit of 2° C, and representative isolates have some resistance to antibiotics [81]. Therefore, they may be well adapted to compete and grow in the melting snowpack, especially considering their faster growth rates [82]. Bacteroidetes were also part of the shift in bacterial community composition, and while they have often been considered r-strategists [22, 67], it has recently been suggested that they should actually be classified as K-strategists [68]. Often associated with algal blooms [80], their relative abundance first decreased before a subsequent increase, a pattern similar to that identified by Lutz et al. [77] and positively correlated with the increased concentration of dissolved organic carbon due to algal blooms [77], providing further evidence to suggest that an algal bloom may have occurred during snow melt. While the shift in snow community composition might result from atmospheric deposition from the phytoplankton bloom occurring in Kongsfjorden at the same time and reflected by the increase in Oxalobacteraceae in the atmosphere [51], we cannot exclude the possibility of an algal bloom also occurring as the snow melted. A simultaneous decrease in Cyanobacteria relative abundance was observed (primarily classified as *Nostoc*) as the abundance of Betaproteobacteria, Bacteroidetes, Chlorophyta, and Streptophyta increased. The decrease of Cyanobacteria may be due to the competition with other organisms, and in this case, heterotrophic bacteria, as has been previously reported in laboratory experiments [83, 84]. Indeed, as available organic matter increases, heterotrophic bacteria grow faster and use the limited phosphate resources, leaving the Cyanobacteria phosphate limited [83].

The large taxonomic shifts in bacterial populations observed on day 15 in the surface snow and from day 11 onward in bulk snow may have resulted from changes in environmental conditions. Increased temperatures, liquid water, and nutrient availability may have promoted the growth of certain taxa such as Betaproteobacteria (Oxalobacteraceae) and Bacteroidetes (Cytophagia, Flavobacteriia, and Sphingobacteria). The shift in taxonomy was reflected by some changes in the functional profile, essentially by an increase in iron acquisition, membrane transport, virulence, motility, and stress-associated genes, potentially influencing the colonisation potential by playing a role in competition, adaptation and survival.

### Resident Community

The major changes observed in the snow eukaryotic communities over time were not reflected in the soil eukaryotic community. The soil bacterial community was dominated by Acidobacteria and Proteobacteria (mainly Alphaproteobacteria and Betaproteobacteria). The 16S profile showed a peak in Firmicutes relative abundance on day 4, driven by the increase of Clostridia, commonly identified in Arctic soils [85, 86] and considered r-strategists with fast growth rates [22, 67], which may be favoured by the changing environmental conditions and out-compete some taxa. Overall, both the taxonomic and functional profiles remained relatively stable during the snow melt. In this study, the experiment stopped on the last day of the melt. During the snow melt, the soil community is still covered by the snowpack, remaining isolated from changes in environmental conditions such as increasing light and temperature. This may explain the low variation in soil communities observed. Soil communities have been shown to change after the melt as they become exposed to changing environmental conditions, plant growth, and increasing activity from macroorganisms [87–89]. Furthermore, the low cell concentration in the snow compared to the soil [90] may have limited the detection of changes in community, especially if the deposited organisms died rapidly.

### Colonisation Potential

A successful colonisation depends on the alpha diversity, ability to survive and compete of both, the invading and resident community [4]. The invaders need to be well adapted to the environmental conditions of the new ecosystem they are attempting to colonise. In this case, the snow microbial communities already lived in similar abiotic conditions, although with its share of differences (e.g. carbon availability or photochemistry), they should have already been better adapted to the soil ecosystem than microorganisms coming from elsewhere, increasing their chances of successful colonisation [4, 5]. Environmental resistance genes were identified such as cobalt-zinc-cadmium resistance which may help adapt to the changing conditions. Furthermore, in the snow, we observed an increase in stress response genes such as bacterial haemoglobins and glutathione-related genes, both involved in the protection to oxidative stress [91, 92]. Osmotic stress was also likely occurring as we identified an increase in ectoine and betaine biosynthesis genes, both protecting against salinity, temperature or osmotic stress [60].

The invaders also need to be good competitors, which is particularly difficult to evaluate in natural, complex ecosystems such as soils. Genes involved with competition such as increased motility and virulence (such as adhesion) were observed. We also identified antibiotic resistance genes as well as resistance to fluoroquinolones and beta-lactamase genes. The increase of these genes in the snow communities may provide a competitive advantage by increasing the potential for a successful colonisation of the soil. The functional profile of the soil remained relatively stable over time with no apparent increase in competition genes. This may reflect the low pressure exerted by the invaders on the soil community, which may already be well protected by the stability and cellular abundance, limiting the risks of invasions.

While 596 ASVs were initially present in the snow only and later identified in the soil, 100 ASVs were considered potentially successful colonisers. Interestingly, the majority of these colonisers were considered K-strategists [22, 67] and is against the hypothesis that r-strategists are more likely to colonise due to faster growth rates [17]. K-strategists may have the advantage in the Arctic as they may be better adapted to survive stressful environmental condition and resource limitations [21, 22]. We should note that characterising life-strategy based on taxonomy remains difficult and largely inaccurate, highlighting the need for further fundamental research.

Overall, this study provides the first insights into the impact of snow melt on soil microbial communities. While we did record some likely invasions, we did not assess whether these potentially successful colonisers survived in the long run and the impact they may have had on the soil community. Laboratory microcosm experiments suggested that 15 days after the end of melt, only few ASVs successfully colonised the soil, primarily classified as r-strategists [14]. Therefore, whether these 100 ASVs were successful remains undetermined and while K-strategists may be favoured after the melt, r-strategists may be more successful in the long run. Furthermore, we should note that due to limited sequencing depth, the genes identified are likely coming from the most abundant organisms in the community investigated [33, 93]. These results are providing indications that colonisation likely occurs following snow melt but monitoring communities throughout the melt and the summer season, with increased replication of the sampling design and deeper sequencing should be conducted to assess whether invaders can permanently colonise and grow into the soil community and their impact on resident communities.

## Supplementary Information

Below is the link to the electronic supplementary material.

Supplementary file1 (DOCX 1.65 MB)

## Data Availability

The 16S rRNA dataset is deposited at the European Nucleotide Archive under the BioProject accession PRJNA564220. The shotgun metagenomics dataset is deposited on MG-RAST under the accession mgp89221.

## Code Availability

All codes for analysis can be obtained by contacting the corresponding author.
