# Supplementary material for: Snow Microorganisms Colonise Arctic Soils Following Snow Melt
Source: Microb Ecol. 2023 Mar 20;86(3):1661–75. doi: 10.1007/s00248-023-02204-y (PMC10497451; doi:10.1007/s00248-023-02204-y)
Supplement: Supplementary file 1 — Supplementary file1 (DOCX 1.65 MB) [file 248_2023_2204_MOESM1_ESM.docx]

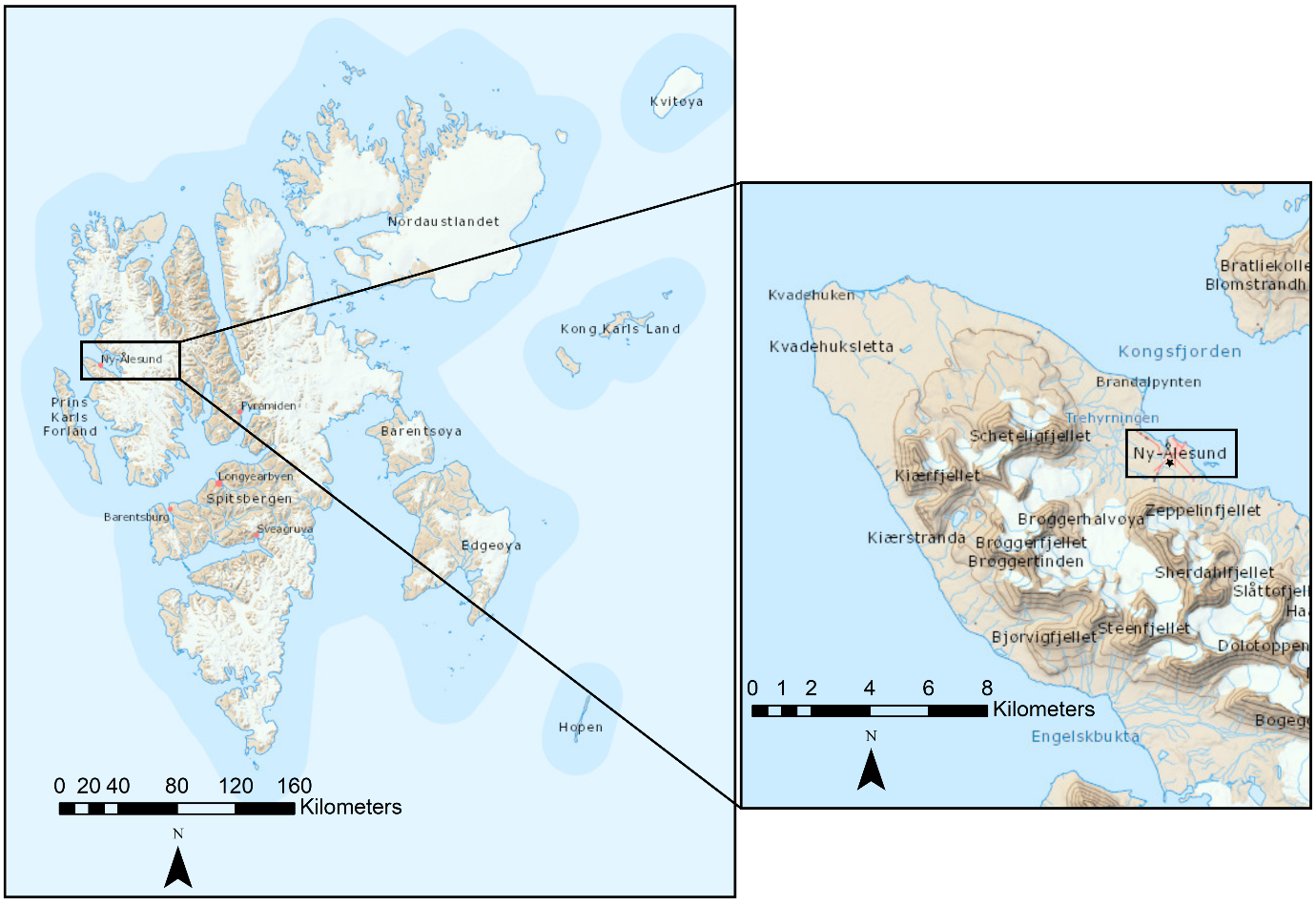


***Figure S1:*** *Map showing the localisation of Ny-Alesund, the sampling site.*


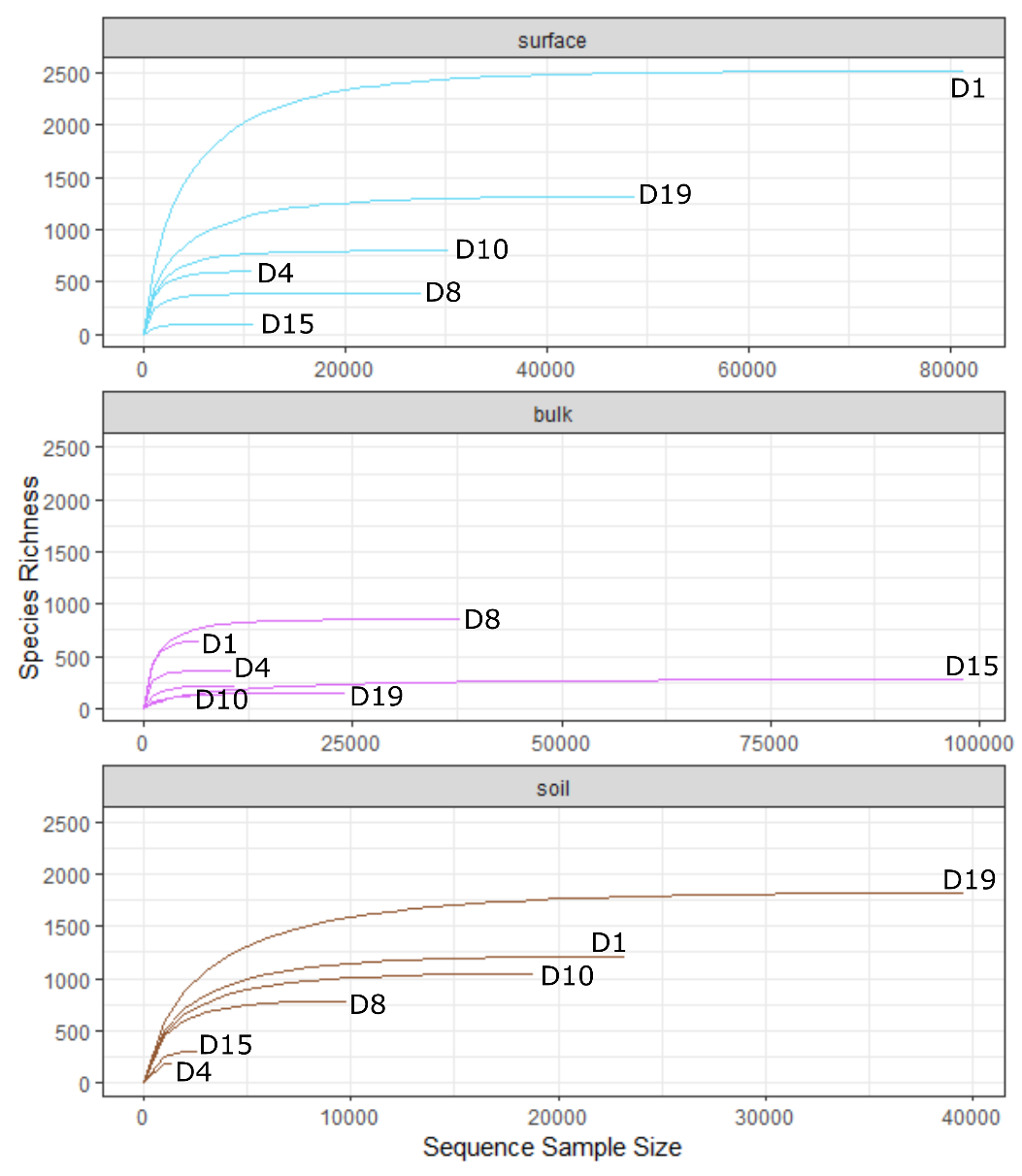


***Figure S2:*** *Rarefaction curves by sample type and sampling day.*


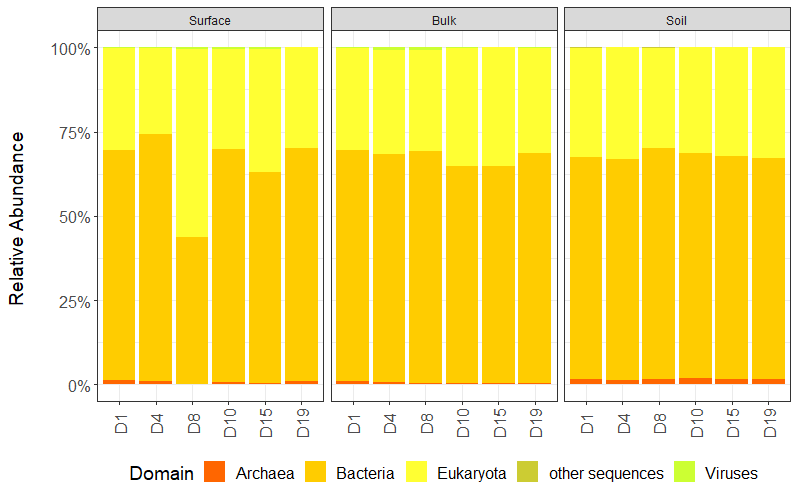


***Figure S3:*** *Taxonomic composition of the shotgun metagenomic library at the domain level illustrating the dominance of bacteria and eukaryotes in the microbial communities.*


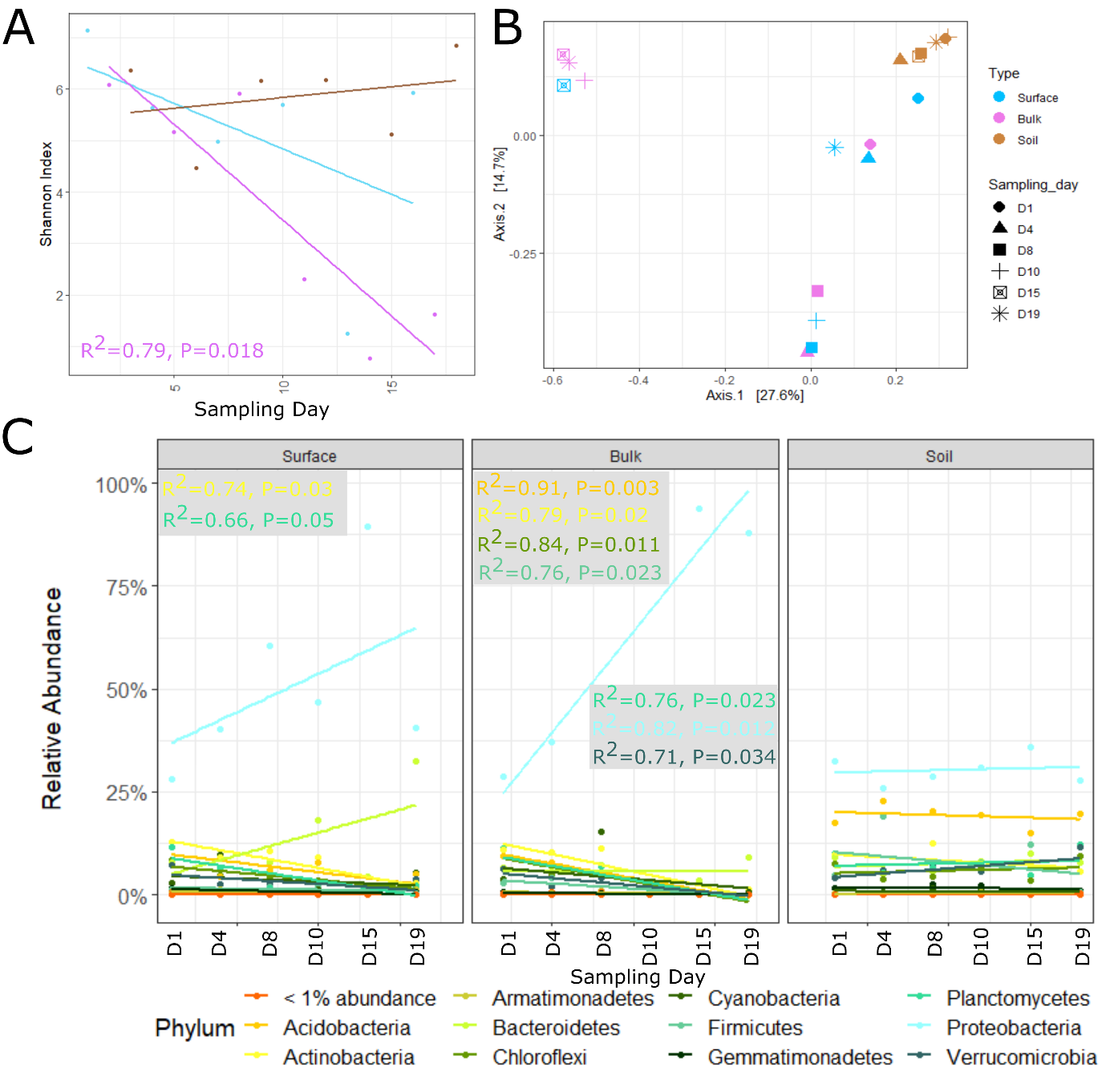


***Figure S4:*** Bacterial communities assessed via 16S amplicon sequencing. Significant (p<0.05) linear regressions are displayed. (A) Bacterial diversity at each sampling day. (B) PCoA of the bacterial communities showing the stability of soil communities, the two clusters of bulk snow and the variability of surface snow communities. (C) Changes in bacterial community composition at the phylum level.

***Table S1: Sample characteristics and MG-RAST accession numbers with sequence count.***

| **Sample name** | **Sample day** | **Sample date** | **MG-RAST accession** | **Type** | **Shotgun metagenomics sequence count** | **16S sequence count** |
| --- | --- | --- | --- | --- | --- | --- |
| 1.1Surface | D1 | 01/05/2018 | mgs736702 | Surface | 132,265 | 81176 |
| 1.2Bulk | D1 | 01/05/2018 | mgs736705 | Bulk | 20,952 | 6535 |
| 1.3Soil | D1 | 01/05/2018 | mgs736708 | Soil | 160,755 | 22897 |
| 2.1Surface | D4 | 04/05/2018 | mgs736717 | Surface | 268,616 | 10537 |
| 2.2Bulk | D4 | 04/05/2018 | mgs736714 | Bulk | 106,321 | 10558 |
| 2.3Soil | D4 | 04/05/2018 | mgs736720 | Soil | 100,022 | 1327 |
| 3.1Surface | D8 | 08/05/2018 | mgs736723 | Surface | 16,365 | 27147 |
| 3.2Bulk | D8 | 08/05/2018 | mgs736726 | Bulk | 18,519 | 37959 |
| 3.3Soil | D8 | 08/05/2018 | mgs736729 | Soil | 248,583 | 9527 |
| 4.1Surface | D11 | 11/05/2018 | mgs736732 | Surface | 38,230 | 30115 |
| 4.2Bulk | D11 | 11/05/2018 | mgs736735 | Bulk | 183,740 | 10861 |
| 4.3Soil | D11 | 11/05/2018 | mgs736738 | Soil | 282,257 | 18447 |
| 5.1Surface | D15 | 15/05/2018 | mgs736741 | Surface | 33,602 | 10860 |
| 5.2Bulk | D15 | 15/05/2018 | mgs736744 | Bulk | 290,789 | 98084 |
| 5.3Soil | D15 | 15/05/2018 | mgs736747 | Soil | 232,778 | 2522 |
| 6.1Surface | D19 | 19/05/2018 | mgs736750 | Surface | 312,882 | 48480 |
| 6.2Bulk | D19 | 19/05/2018 | mgs736753 | Bulk | 98,161 | 23990 |
| 6.3Soil | D19 | 19/05/2018 | mgs736756 | Soil | 234,623 | 39133 |

***Table S2: Classification of potential colonists based on the literature as either copiotrophs (r-strategists), oligotrophs (K-strategists) or unclear (when no information was found).***

| ***Kingdom*** | ***Phylum*** | ***Strategy*** | ***Copio-Oligo*** | ***Reference*** |
| --- | --- | --- | --- | --- |
| *Acidobacteria* | *Blastocatellia* | *K* | *Oligotroph* | *[1, 2]* |
| *Acidobacteria* | *Holophagae* | *K* | *Oligotroph* | *[1, 2]* |
| *Acidobacteria* | *Subgroup 17* | *K* | *Oligotroph* | *[1, 2]* |
| *Acidobacteria* | *Subgroup 6* | *K* | *Oligotroph* | *[1, 2]* |
| *Actinobacteria* | *Acidimicrobiia* | *r* | *Copiotroph* | *[2, 3]* |
| *Actinobacteria* | *Actinobacteria* | *r* | *Copiotroph* | *[2, 3]* |
| *Actinobacteria* | *Thermoleophilia* | *r* | *Copiotroph* | *[2, 3]* |
| *Armatimonadetes* | *Armatimonadia* | *Unclear* | *Unclear* |  |
| *Bacteroidetes* | *Cytophagia* | *K* | *Oligotroph* | *[2, 3]* |
| *Bacteroidetes* | *Sphingobacteriia* | *K* | *Oligotroph* | *[2, 3]* |
| *Chlorobi* | *Chlorobia* | *Unclear* | *Unclear* |  |
| *Chloroflexi* | *Anaerolineae* | *K* | *Oligotroph* | *[2, 3]* |
| *Chloroflexi* | *KD4-96* | *K* | *Oligotroph* | *[2, 3]* |
| *Cyanobacteria* | *Melainabacteria* | *K* | *Oligotroph* | *[2, 3]* |
| *Elusimicrobia* | *Elusimicrobia* | *Unclear* | *Unclear* |  |
| *Firmicutes* | *Clostridia* | *r* | *Copiotroph* | *[2]* |
| *Planctomycetes* | *OM190* | *K* | *Oligotroph* | *[2, 3]* |
| *Planctomycetes* | *Phycisphaerae* | *K* | *Oligotroph* | *[2, 3]* |
| *Planctomycetes* | *Planctomycetacia* | *K* | *Oligotroph* | *[2, 3]* |
| *Proteobacteria* | *Alphaproteobacteria* | *r* | *Copiotroph* | *[2]* |
| *Proteobacteria* | *Betaproteobacteria* | *r* | *Copiotroph* | *[1, 2]* |
| *Proteobacteria* | *Deltaproteobacteria* | *K* | *Oligotroph* | *[2, 3]* |
| *Proteobacteria* | *Gammaproteobacteria* | *r* | *Copiotroph* | *[2]* |
| *Verrucomicrobia* | *OPB35_soil_group* | *K* | *Oligotroph* | *[2]* |
| *Verrucomicrobia* | *Opitutae* | *K* | *Oligotroph* | *[2]* |
| *Verrucomicrobia* | *Spartobacteria* | *K* | *Oligotroph* | *[2]* |
| *Verrucomicrobia* | *Verrucomicrobia_Incertae_Sedis* | *K* | *Oligotroph* | *[2]* |

***References for the classification of copiotrophic/oligotrophic lifestyles***

1. Fierer N, Bradford MA, Jackson RB (2007) Toward an ecological classification of soil bacteria. Ecology 88: 1354-1364.

2. Ho A, Di Lonardo DP, Bodelier PL (2017) Revisiting life strategy concepts in environmental microbial ecology. FEMS microbiology ecology 93: fix006.

3. Finn DR, Bergk-Pinto B, Hazard C, Nicol GW, Tebbe CC, Vogel TM (2021) Functional trait relationships demonstrate life strategies in terrestrial prokaryotes. FEMS Microbiology Ecology 97: fiab068.
